# Long-term survival in a male patient with Müllerian adenocarcinoma of the pelvis: a 15-year case report

**DOI:** 10.3389/fonc.2025.1610783

**Published:** 2025-09-02

**Authors:** Girolamo Ranieri, Mariangela Porcelli, Alessandro Mastrorosa, Alessandra Di Palo, Cristina Ferrari, Alfredo Francesco Zito, Michele Ammendola, Carmelo Laface, Giovanni Mastrandrea, Simona Berardone

**Affiliations:** ^1^ Unità Operativa di Oncologia Integrata, IRCCS Istituto Tumori “Giovanni Paolo II”, Bari, Italy; ^2^ Unità Operativa di Oncologia Urologica, IRCCS Istituto Tumori “Giovanni Paolo II”, Bari, Italy; ^3^ Unità Operativa di Medicina Nucleare,Università di Bari “Aldo Moro”, Bari, Italy; ^4^ Unità Operativa di Anatomia Patologica, IRCCS Istituto Tumori “Giovanni Paolo II”, Bari, Italy; ^5^ Unità Operativa di Chirurgia dell'Apparato Digerente, Università “Magna Grecia”, Catanzaro, Italy; ^6^ Unità Operativa Complessa di Anestesia, Rianimazione e Terapia Intensiva Postoperatoria, IRCCS Istituto Tumori “Giovanni Paolo II”, Bari, Italy

**Keywords:** Müllerian adenocarcinoma in male, pelvis, multimodal oncology therapy, long survival, case report

## Abstract

**Background:**

Müllerian adenocarcinoma is a very rare and aggressive cancer originating from the uterus and ovary, affecting almost exclusively women. Few cases of extragenital Müllerian adenocarcinomas have been reported, in various locations ranging from pelvic peritoneum to diaphragm peritoneum. Very few cases of Müllerian adenocarcinoma in men have been reported in scientific literature, usually localized in the prostate seminal vesicles and testicles, associated to a very poor prognosis.

**Case summary:**

In this paper, we describe the unique clinical case of a man affected by advanced Müllerian adenocarcinoma on the left side of the pelvis, treated with a combination of surgery, chemotherapy, and radiotherapy, with a long follow-up. Patient survival was exceptional (almost 15 years from diagnosis), and the patient experienced a good quality of life during the numerous treatments. To the best of our knowledge, this is the first case report regarding a Müllerian adenocarcinoma of the pelvis in a man treated with a multimodal therapy approach and with a very long follow-up and very long survival. In consideration of the embryological origin of ovaries from Müllerian ducts and the absence of specific guidelines for standard treatment for this tumor, the patient was treated as if he had ovarian cancer, with optimal results.

**Conclusions:**

The management of this patient with modern available lines of chemotherapy classically employed in ovarian cancer plus radiotherapy combined with several bouts of cytoreductive surgery could explain this long survival.

## Introduction

Müllerian adenocarcinoma is a very rare and aggressive cancer originating from the uterus and ovary (1), predominantly affecting women. Its rarity is clearly related to the absence of a female embryonic component in men. It is well known that there is an undifferentiated gonad in the initial stages of embryogenesis. Therefore, if there is a differentiation in the male direction, the female embryonic component goes into complete involution. Conversely, if there is a differentiation in the female direction, the male gonad goes into complete regression. The pathogenetic hypothesis of the Müllerian tumor in humans refers to clusters of female Müllerian cells that have not completely regressed. Müllerian tumor cells could be created from these clusters.

Few cases of extragenital Müllerian adenocarcinomas have been reported, in various locations ranging from pelvic peritoneum to diaphragm peritoneum (1). Very few cases of Müllerian adenocarcinoma in men have been reported in scientific literature, usually localized in the prostate seminal vesicles and testicles, associated to a very poor prognosis (2–4).

In this paper, we describe the unique clinical case of a man aged between 60 and 80 years and affected by advanced Müllerian adenocarcinoma on the left side of the pelvis, treated with a combination of surgery, chemotherapy, and radiotherapy, with a long follow-up (5, 6).

Patient survival was exceptional (almost 15 years from diagnosis), and the patient experienced a good quality of life (QoL) during the numerous treatments. To the best of our knowledge, this is the first case report regarding a Müllerian adenocarcinoma of the pelvis in a man treated with a multimodal therapy approach (surgery plus chemotherapy plus radiotherapy) with a very long follow-up and very long survival (5, 6).

The therapeutic strategy employed for this patient, including chemotherapy and surgical treatments, might represent an important reference for the treatment of these very rare and aggressive tumors in men.

## Case presentation

**Initial presentation:** A male patient aged between 60 and 80 years presented with a weight of 70 kg, a height of 175 cm, and a BMI of 22.86, suffering from a left inguinal hernia that was never surgically treated.

In the first half of 2005, he was hospitalized at the Surgery Unit of Di Venere Hospital of Bari (Italy) for resection surgery of the sigma and placement of a ureteral stent because of an intestinal occlusion and hydronephrosis of the left kidney due to a mass of 4 cm at the left side of pelvis with infiltration of the left ureter and the sigma. He was married and a father of children. He was normally virilized and had normal genitals and mild gynecomastia.

**Figure 1:**
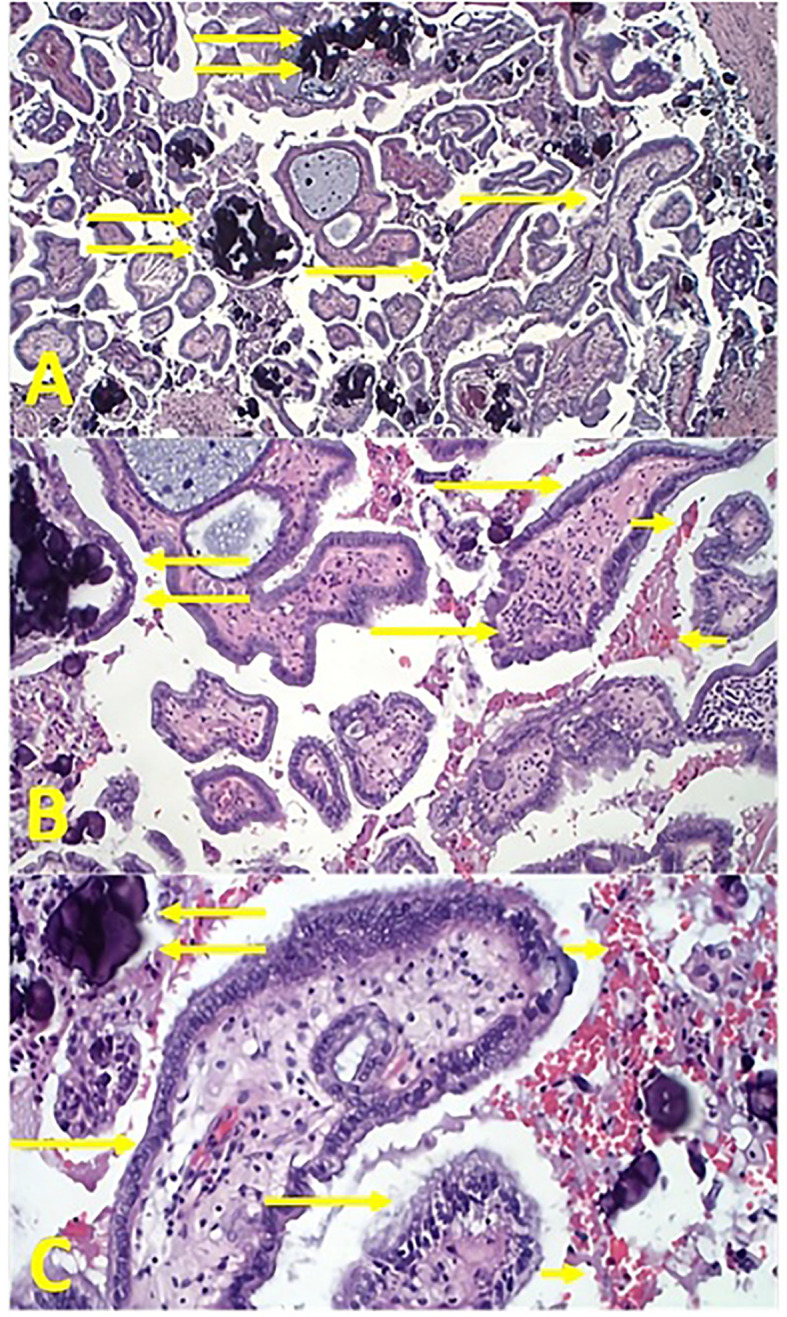
Tumor sections stained with hematoxylin and eosin technique: an overview of a typical papillary serous carcinoma of the ovary. **(A)** Low magnification ×100 in light microscopy. Single arrows indicate typical papillae of Müllerian cancer and the double arrow indicates the characteristic dark psammomatous bodies, which can also be macroscopically observed in CT and PET scans (**Figure 3**). **(B)** Magnification ×200 in light microscopy. Single arrows indicate typical papillae of Müllerian cancer; the double arrow indicates the characteristic dark psammomatous bodies, which can also macroscopically be observed in CT and PET scans; the small arrow indicates blood extravasations. **(C)** Magnification ×400 in light microscopy. Single arrows indicate two papillae of Müllerian cancer with clear epithelial pluristratification and failure to respect the basement membrane with stromal infiltration; the double arrow indicates the characteristic dark psammomatous bodies, which can also be macroscopically observed in CT and PET scans; the small arrow indicates blood extravasations.

**Histopathological findings:** The first histopathological examination was not conclusive, and a second histopathological opinion has been performed at IRCCS Istituto Tumori *Giovanni Paolo II* of Bari (Italy). Upon examination using the hematoxylin and eosin technique, epitheliomorphic cancer was diagnosed. It consisted of adeno-papillary architecture with cells expressing a ciliated border and with a rich psammomatous stromal component (**Figures 1A–C**) (7).

**Figure 2:**
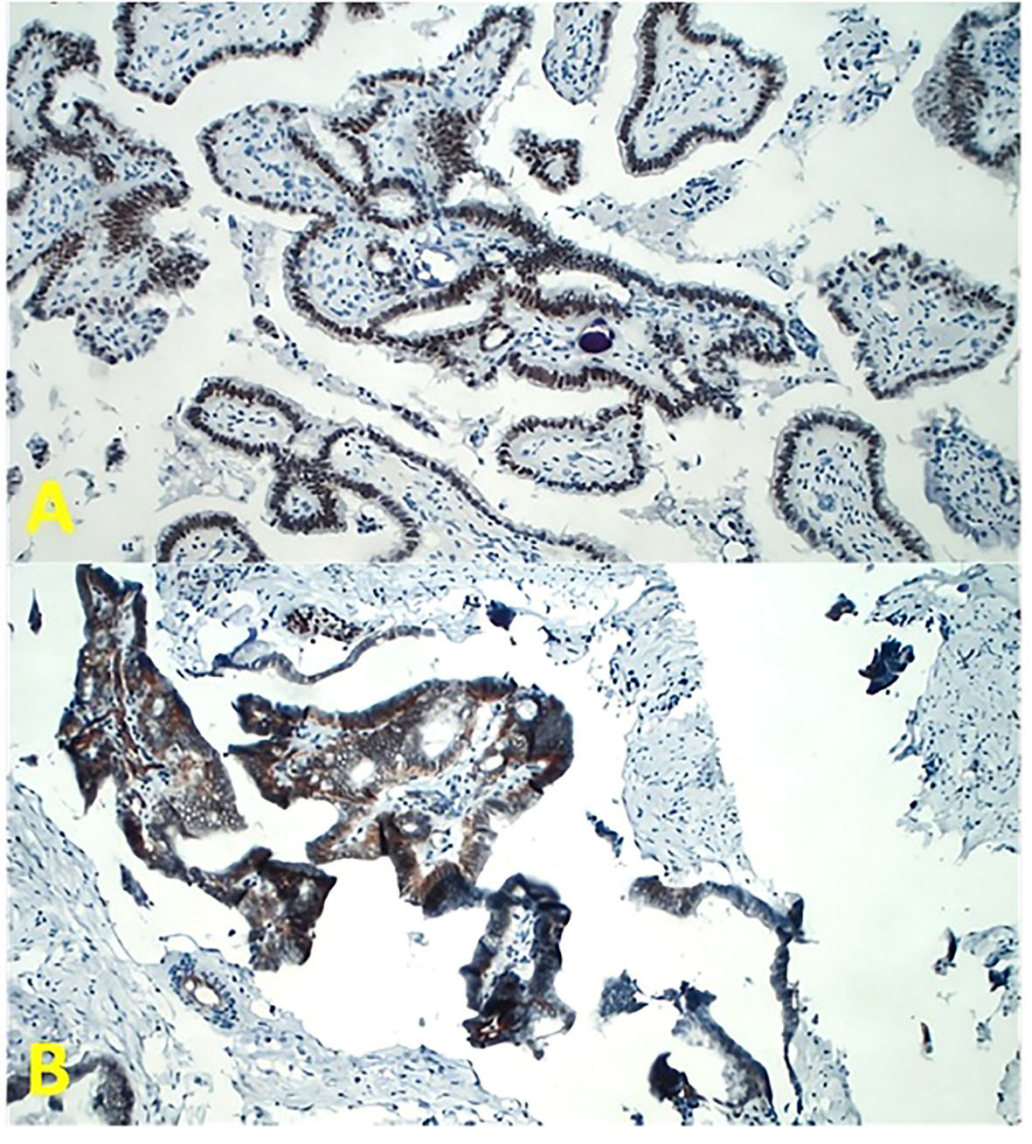
Tumor sections for immunohistochemistry at magnification ×200 in light microscopy. **(A)** The antibodies for estrogen receptor (ER) (Dako Flex Monoclonal Rabbit Anti-Human ER α, Clone EP1) and progesterone receptor (PgR) (Dako Monoclonal Mouse Anti-Human Progesterone Receptor Clone PgR 636) have been employed. Note the strong nuclear brown immunoreactivity (diaminobenzidine chromogen). **(B)** The antibody for Wilms’ Tumor One (WT1) receptor has been employed (Dako Flex Monoclonal Mouse Anti-Human WT1 Protein Clone 6F-H2). Note the strong membrane immunoreactivity for WT1.

Immunohistochemical examination demonstrated a strong nuclear brown immunoreactivity for estrogen receptors (diaminobenzidine chromogen) with focal and limited immunoreactivity for progesterone receptors (**Figure 2A**).

Finally, immunohistochemistry also showed a strong membrane immunoreactivity for Wilms’ Tumor One (WT1), a typical marker of ovarian cancer. WT1, located on chromosome 11p13, is a tumor suppressor gene involved in Wilms’ tumor development. Normally, its expression is limited to the kidney, gonads, spleen, hematopoietic precursors, and fetal mesothelium. Furthermore, WT1 also exhibits oncogenic activity, and its overexpression has been well demonstrated in ovarian cancer; thus, WT1 immunohistochemical detection is considered as a diagnostic marker of ovarian cancer (**Figure 2B**) (8).

**Diagnosis:** On these bases, the diagnosis of a very rare papillary serous adenocarcinoma originating from Müllerian ducts residues was formulated in this patient.

### Treatment

The patient was offered adjuvant chemotherapy in accordance with the treatment strategy for ovarian cancer. The patient has undergone adjuvant chemotherapy with carboplatin (AUC 5) plus paclitaxel (175 mg/m^2^) every 3 weeks for six cycles at the Oncology Unit of IRCCS *De Bellis* of Castellana Grotte (Italy) (9). Subsequently, follow-up was started including Ca125 serum dosage, thorax–abdomen CT scan every 6 months, and positron emission tomography/computed tomography (PET/CT) scan every 1 year.

### Outcome and follow-up

In the first half of 2011, a radiological evaluation put in evidence a pelvic recurrence of the disease. Total body PET/CT showed radiopharmaceutical hyperaccumulation in the left iliac region [maximum Standard Uptake Value (mSUV) of 9.9], presacral/coccygeal area (mSUV of 4.2), peri-bladder region (mSUV of 8.9), and at the posterior wall of the rectum (mSUV of 3.2). Therefore, he was admitted to the Surgery Unit of IRCCS Istituto Tumori *Giovanni Paolo II* of Bari (Italy) for resection of pelvic disease recurrence. In consideration of tumor platinum sensibility (6 years of disease-free survival from the last administered cycle of platinum-based chemotherapy), the patient received a re-challenge of the adjuvant therapy with carboplatin and paclitaxel at the Integrative Medical Oncology Unit of IRCCS Istituto Tumori *Giovanni Paolo II* of Bari (Italy).

**Figure 3:**
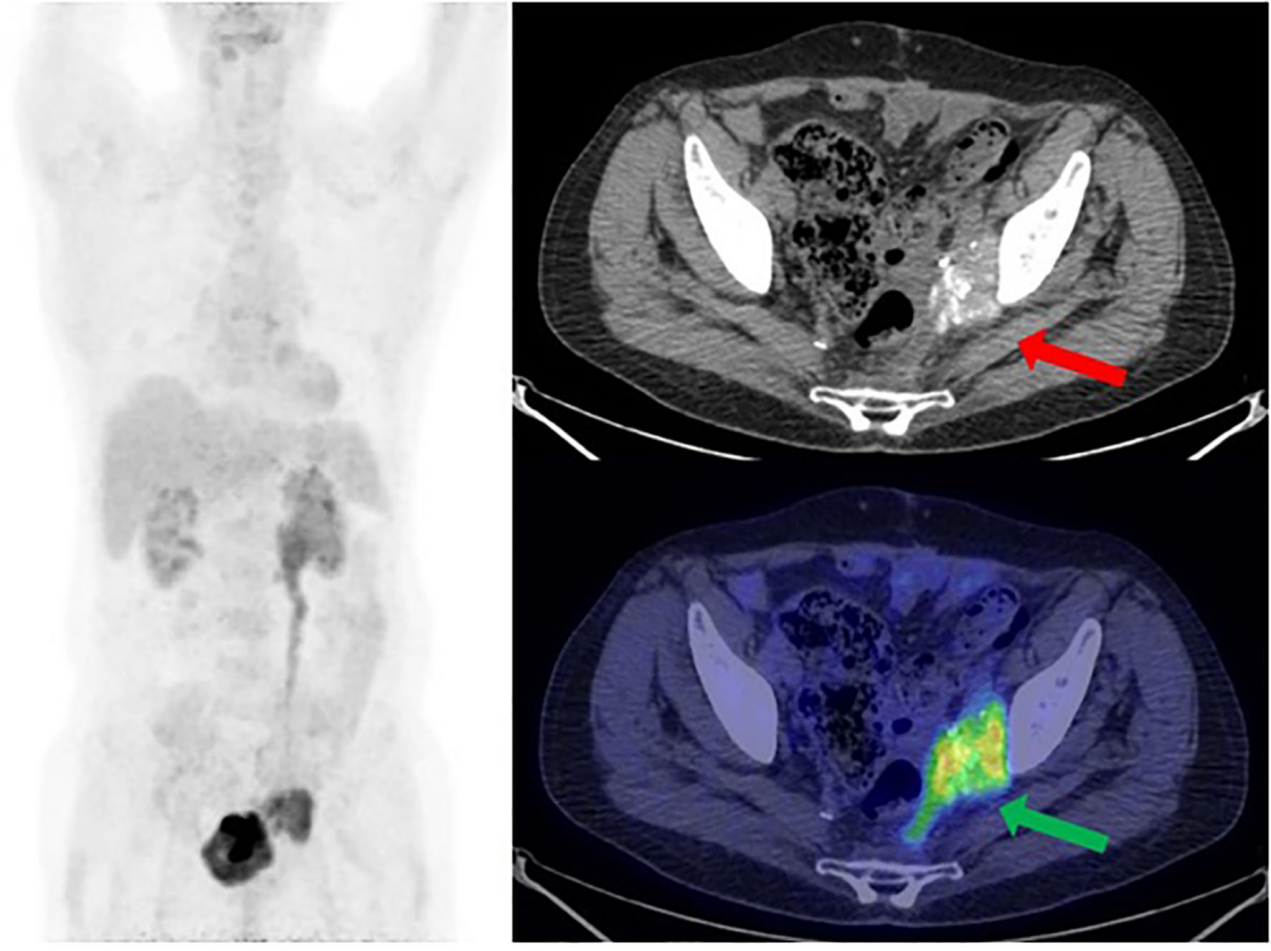
[18F] FDG PET/CT MIP reconstruction and transaxial image (performed at the University Hospital of Bari) showed the recurrence of disease at the left side of the pelvis, near the bladder, with a size of 3.7 × 4.8 cm and an mSUV of 7.7 (green arrow). Note the psammomatous bodies (concentric calcifications, a consequence of apoptotic cell desquamation—red arrow), a typical feature of ovarian tumors, which confirm the Mullerian origin of this man’s tumor.

One year later, a follow-up CT scan shows a left obturator and peri-bladder neoformation of 4 cm. PET/CT documented radiopharmaceutical hyperaccumulation at the left obturator (mSUV of 7.7) (**Figure 3**). These lesions were also evident at CT of co-registration.

In the second half of 2013, 15 cycles of radiotherapy at IRCCS Istituto Tumori *Giovanni Paolo II* of Bari (Italy) are carried out on pelvic recurrence (200 cGy for each day for 3 weeks, 5 days a week) in the left obturator region to reduce painful symptoms localized in the left pelvic region and hip. The radiological and laboratory follow-up exams showed the stability of disease (SD) until December 2013. In consideration of tumor platinum sensibility, 2 years of progression-free survival (PFS) from the last administered cycle of platinum-based chemotherapy, the patient again received chemotherapy with carboplatin and paclitaxel at the Integrative Medical Oncology Unit of IRCCS Istituto Tumori *Giovanni Paolo II* of Bari (Italy). The patient experienced a good QoL and a PFS of 3 years.

**Figure 4:**
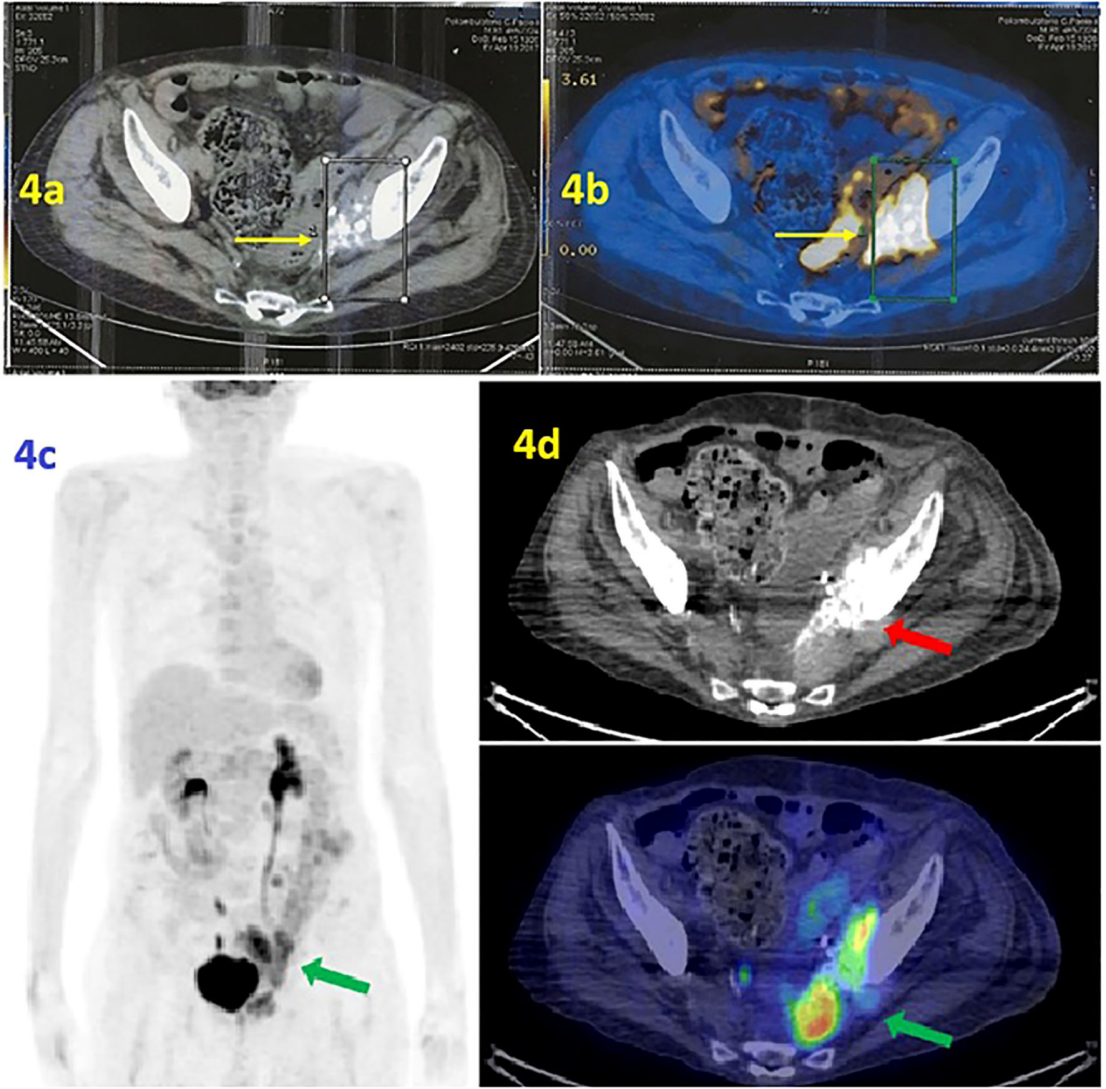
**(a, b)** PET scan/CT scan. **(a)** The PET scan (performed at IRCCS *Casa Sollievo della Sofferenza* Foundation in April 2017) shows radiopharmaceutical hyperaccumulation in the left area of the pelvis with an mSUV of 10.1. **(b)** Co-registration CT scan documented the volumetric increase of lesion. Note the psammomatous bodies, a typical marker of ovarian tumors such as adenocarcinoma of Mullerian origin. **(c, d)** [18F] FDG PET/CT MIP reconstruction and transaxial images (performed at the University Hospital of Bari in June 2019) showed the presence of a tumor lesion in the area near the left side of the bladder, adjacent to the obturator muscle and extended anteriorly to the bladder with an mSUV of 12.5 (green arrow). The psammomatous bodies are evident, a marker of ovarian tumors that confirms the tumor’s Mullerian origin (red arrow).

In the first half of 2017, the PET/CT scan showed a progression of disease (PD) consisting of increased uptake of radiopharmaceuticals (**Figure 4a**). At the same time, the co-registration CT scan documented the volumetric increase of the tumoral mass, from 4 to 6 cm, at the left perivesical site of the pelvis. Please note the characteristic psammomatous bodies in the lesion (**Figure 4b**).

Therefore, the second line of chemotherapy with carboplatin AUC 3.5 mg/mL per minute plus gemcitabine 800 mg/m^2^ was administered at the Integrative Medical Oncology Unit of IRCCS Istituto Tumori *Giovanni Paolo II* of Bari (Italy). Thus, the patient experienced a PFS of 3.5 years. In June 2019, PET/CT showed PD due to an increased radiopharmaceutical uptake at the target lesion (mSUV of 12.5) (**Figures 4c, d**).

On this basis, chemotherapy with gemcitabine 800 mg/m^2^ for 12 weekly cycles was administered until January 2020 for further PD.

Unfortunately, the patient died in 2020 because of a septic shock.

## Discussion

The tumor that originates from residues of Müllerian ducts is very rare in women and exceptional in men, with a poor prognosis. Because of its extreme rarity, the diagnosis is not often considered, and no guidelines establishing any standard treatment are available.

In this case report, we described the clinical history of a male patient affected by a Müllerian tumor with a 15-year survival from diagnosis to death, thanks to a treatment strategy including surgery, several chemotherapy lines, and radiotherapy (**Table 1**) (9, 10). It should be noted that the WT1 antibody was employed for histopathological examination. The strong membrane immunoreactivity to WT1 antibody suggests its possible use as a marker for diagnosis (8).

**Table 1:** Chronological summary of treatments and outcomes.

Date	Treatment	Response
2005	Surgical resection of sigmoid colon and ureteral stent placement due to pelvic mass with ureter and sigmoid infiltration, and adjuvant chemotherapy: Carboplatin (AUC 5) + paclitaxel (175 mg/m²) × six cycles	CR
2011	Surgery for pelvic recurrence and subsequent chemotherapy with carboplatin + paclitaxel	PR
2013	Pelvic radiotherapy (15 cycles, 200 cGy/day × 3 weeks) for local recurrence (left obturator, peri-bladder) and re-challenge with carboplatin + paclitaxel	PD
2017	Second-line chemotherapy: carboplatin AUC 3.5 + gemcitabine 800 mg/m², because of progression on PET/CT (lesion growth and increased uptake)	PD
2019	Gemcitabine 800 mg/m² × 12 weekly cycles (progression on PET/CT)	PD
2020	Death due to septic shock	–

CR, complete response; PR, partial response; SD, stable disease; PD, progressive disease.

In consideration of the embryological origin of ovaries from Müllerian ducts and the absence of specific guidelines for standard treatment for this tumor, the patient was treated as if he was affected by ovarian cancer, with optimal results. Our experience also suggests the Müllerian tumor sensitivity to radiotherapy.

Interestingly, the survival of our patient was impressive, especially if compared to other cases (10–12). The possible explanation for this favorable prognosis could be related to the low production of estrogens in men as compared to women; this hypothesis could be supported by the absence of the persistent Müllerian duct syndrome in our patient, although the tumor expressed estrogen receptors.

Based on our literature search, there do not appear to be any studies that have correlated estrogen levels with the prognosis of male Müllerian carcinoma. We found only 3 case reports of male Müllerian carcinoma with a low survival rate (**Table 2**). Therefore, the rarity of the event may justify this lack of data. However, some studies have hypothesized that high estrogen levels may be related to the development of ovarian cancer. In addition, it is known that some studies indicate that hormonal therapies that inhibit the estrogen–estrogen receptor axis (e.g., tamoxifen) could be a therapeutic approach in female ovarian cancer (13).

**Table 2:** Comparable cases.

N	Title	Authors	Source	Year	Patient	Therapy	Overall Survival
1	Papillary serous carcinoma of the peritoneum in a man: a case report	Shah IA, Jayram L, Gani OS, Fox IS, Stanley TM.	Cancer	1998	74 years old man	cisplatin (HIPEC) and paclitaxel (iv)	3 mounth
2	Primary papillary serous carcinoma of the peritoneum in a man	Shmueli E, Leider-Trejo L, Schwartz I, Aderka D, Inbar M.	Ann Oncol	2001	53 years old man	cisplatin and 5-fluorouracil (iv)	2 mounth
3	Photodynamic detection and management of intraperitoneal spreading of primary peritoneal papillary serous carcinoma in a man: report of a case	Canbay E, Ishibashi H, Sako S, Kitai T, Nishino E, Hirano M, Mizumoto A, Endo Y, Ogura S, Yonemura Y.	Surg Today	2014	63 years old man	cytoreductive surgery and docetaxel (HIPEC) and docetaxel (LPS) and cisplatin (iv)	18 mounths

In addition, the management of this patient with modern available lines of chemotherapy classically employed in ovarian cancer, plus radiotherapy combined with several bouts of cytoreductive surgery, could explain this exceptional long survival (14–16).

Finally, the data reported in this paper might be useful in defining the diagnosis of Mullerian tumor for the suspected clinical cases and the management of these patients.

## Data Availability

The original contributions presented in the study are included in the article/supplementary material. Further inquiries can be directed to the corresponding author.
